# RepeatsDB in 2025: expanding annotations of structured tandem repeats proteins on AlphaFoldDB

**DOI:** 10.1093/nar/gkae965

**Published:** 2024-10-30

**Authors:** Damiano Clementel, Paula Nazarena Arrías, Soroush Mozaffari, Zarifa Osmanli, Ximena Aixa Castro, RepeatsDB curators, Estefanía Lorena Borucki, Maia Cabrera, Patricio Chinestrad, Ian Czarnowski, Jose Francisco Lombardo, Pablo Lorenzano Menna, Ezequiel Gerardo Mogro, Carla Luciana Padilla Franzotti, Julia Yamila Santillan, Carlo Ferrari, Andrey V Kajava, Silvio C E Tosatto, Alexander Miguel Monzon

**Affiliations:** Department of Biomedical Sciences, University of Padova, Padova, Italy; Department of Protein Science, KTH Royal Institute of Technology, Stockholm, Sweden; Department of Biomedical Sciences, University of Padova, Padova, Italy; Department of Biomedical Sciences, University of Padova, Padova, Italy; Department of Biomedical Sciences, University of Padova, Padova, Italy; Department of Information Engineering, University of Padova, Padova, Italy; Centre de Recherche en Biologie Cellulaire de Montpellier, CNRS, Universite′ Montpellier, Montpellier, France; Department of Biomedical Sciences, University of Padova, Padova, Italy; Institute of Biomembranes, Bioenergetics and Molecular Biotechnologies, National Research Council (CNR-IBIOM), Bari, Italy; Department of Information Engineering, University of Padova, Padova, Italy

## Abstract

RepeatsDB (URL: https://repeatsdb.org) stands as a key resource for the classification and annotation of Structured Tandem Repeat Proteins (STRPs), incorporating data from both the Protein Data Bank (PDB) and AlphaFoldDB. This latest release features substantial advancements, including annotations for over 34 000 unique protein sequences from >2000 organisms, representing a fifteenfold increase in coverage. Leveraging state-of-the-art structural alignment tools, RepeatsDB now offers faster and more precise detection of STRPs across both experimental and predicted structures. Key improvements also include a redesigned user interface and enhanced web server, providing an intuitive browsing experience with improved data searchability and accessibility. A new statistics page allows users to explore database metrics based on repeat classifications, while API enhancements support scalability to manage the growing volume of data. These advancements not only refine the understanding of STRPs but also streamline annotation processes, further strengthening RepeatsDB’s role in advancing our understanding of STRP functions.

## Graphical Abstract

**Graphical Abstract:**
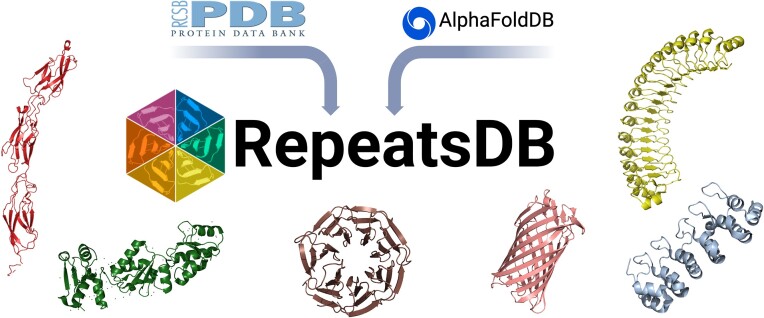

## Introduction

Tandem repeat proteins (TRPs) are a diverse class of proteins characterized by the repetition of specific sequence motifs (1). A subset within TRPs, known as structured tandem repeat proteins (STRPs), is characterized for preserving specific structural motifs, in the absence of sequence similarity (2). STRPs are a specialized subset of TRPs distinguished not only by their sequence repetition but also by their structural characteristics. Defined by regular secondary structure elements and discernible tertiary structures, STRPs may integrate into larger molecular assemblies. According to Kajava's classification (3), most STRPs fall into Classes III (elongated) and IV (closed). However, they can also be found in other classes such as aggregates, fibrous, and bead-on-string repeats. STRPs play pivotal roles in various biological processes and mechanisms. For example, leucine-rich repeats (LRRs) are crucial components of the extracellular domains of Toll-like receptors, which activate host immune responses (4), while STRPs such as DNA sliding clamps are essential in DNA replication processes (5). Moreover, their ability to interact with numerous and diverse proteins favors their involvement in complex formation mechanisms, contributing to the regulation of expression, transcription and splicing (6). The growing focus on TRPs in recent research stems from their crucial roles in health-related studies (7,8) and their utility in the field of protein engineering (9–11).

The first version of RepeatDB (12) was released over a decade ago, becoming the primary repository for STRPs with high-quality annotations of repeat regions, units, and insertions on experimental protein structures according to Kajava's classification. Subsequent releases in 2016 (12) and 2021 (13) have further enhanced these capabilities, extending the manually curated and automatic annotations, and expanding the classification to comprehensively describe STRPs present in the Protein Data Bank (PDB) (14).

The exponential growth in accurate protein structural data in recent years, mostly driven by state-of-the-art Artificial Intelligence (AI) protein structure prediction methods such as AlphaFold (15), RoseTTAFold (16) and ESM Fold (17), has revolutionized the structural bioinformatics community. This surge presents unique challenges in automatically detecting and classifying STRPs with tools like RepeatsDB-Lite (18), TAPO (19) and RAPHAEL (20). Recently developed, STRPsearch (21) is a rapid and accurate method for detecting STRPs from protein structures. This tool enables quick scanning of thousands of structures, facilitating the detection and classification of repeat regions, and identifying units and insertions.

Here, we introduce the updated version of RepeatsDB, accessible at https://repeatsdb.org. This version is designed to incorporate the latest structural data and advances in TRPs, increasing the number of annotated protein sequences fifteen fold compared to the previous version. Extensive community curation efforts have enhanced the database, identifying specific regions, units, and insertions within PDB experimental structures. Additionally, it features automatic annotations of structural models from SwissProt proteins deposited in AlphaFoldDB (22), using STRPsearch. It includes a complete re-annotation of manually curated annotations in order to guarantee their quality, as well as a revised classification. Furthermore, we have upgraded the website and web server architecture, introducing a dedicated platform for manual annotations of STRPs. This not only allows for a faster review process of manually curated annotations but also improves data accessibility and usability.

## Progress and new features

### Database content

RepeatsDB aims to establish itself as the fundamental repository for the annotation and classification of STRPs, sourced from primary structural data databases, such as the PDB for experimental structures and AlphaFoldDB for structural models. Additionally, continuous efforts in the community focus on identifying and characterizing novel tandem repeat proteins, as well as accurately defining repeat protein families. Recently, RepeatsDB has been included as an external resource in the InterPro (23), providing repeat region annotations and enhancing interoperability between these databases. This collaboration also contributes to refining and characterizing repeat protein family boundaries within Pfam at the sequence level (24) and to better defining repeat units from a structural perspective.

In this new release, the number of entries in RepeatsDB has increased more than fifteen times in terms of protein sequences with repeat annotations. A total of 34 319 unique protein sequences, each identified by different UniProtKB identifiers (25), now feature at least one repeat region detected in PDB and/or AlphaFoldDB (Table 1). By providing online tools for the annotation of STRPs, community curation efforts have been directed towards the review of >15 000 experimentally observed structures from the PDB. Using the new algorithm, STRPsearch (21), over 30 000 repeated regions were automatically extracted from AlphaFoldDB/Swiss-Prot. STRPsearch utilizes a Tri-Unit Library composed of manually curated structures from RepeatsDB and employs the fast and accurate structural alignment method, FoldSeek (26). This tool enables rapid and precise detection and classification of repeat regions, units, and insertions within protein structures.

**Table 1.** RepeatsDB in 2025 data

Source	Annotation type	Proteins	Single-chain structures	Regions	Units
Protein Data Bank	*Reviewed*	4092	15141	16 282	106 408
AlphaFoldDB	*Predicted*	30220	30220	33 207	250 416

‘Proteins’ corresponds to the number of different UniProtKB identifiers mapping to a single PDB chain.

### Classification schema

This updated version of RepeatsDB retains the 4-level classification scheme introduced in version 3.0, which is based on Kajava's class assignment (3) and aims at integrating and maintaining coherence with the Pfam database. While no new topologies have been introduced in this update, two existing topologies, namely the ‘Beta trefoil’ and the ‘Alpha/beta trefoil’, have been merged into ‘trefoil’ based on structural clustering.

Several adjustments have been made at the third level, ‘Fold’, to accommodate new data and refine the definition of RepeatsDB ‘Fold’. For instance, two new folds have been created within the ‘Alpha beads’ topology to include ‘Spectrin repeats’ and ‘FF-repeats’. Additionally, two folds have been established in the ‘Beta solenoid’ topology to account for solenoid ‘handedness’, which refers to the direction in which the chain winds around the molecular axis (27).

Significant progress has also been made in ‘Clan’ assignment, largely facilitated by clustering the STRPs based on structural similarity at three hierarchical levels of single unit, tri-unit and region, by DBSCAN (density-based spatial clustering of applications with noise) algorithm of scikit-learn (28). The resulting clusters were analyzed and compared in relation to their corresponding Pfam annotations of their members. This method improved the refinement of clan classifications by enabling the merging of existing clans or their division into new ones. For example, a total of 11 new clans have been assigned across the seven folds of the ‘Propeller’ topology.

### Data generation and updates

#### RepeatsDB biocuration tool

A new feature of this version of RepeatsDB is the dedicated Biocuration tool, developed to manually curate and verify STRPsearch predictions or perform *de novo* annotations on protein structures. The RepeatsDB Biocuration tool offers an easy-to-use user interface that allows biocurators to log in and access STRPsearch predictions stored in the staging database (see the implementation section), or to annotate a protein structure from scratch. It uses the ORCID authentication service to link annotations to a unique researcher identifier. Biocurators can log into the Biocuration tool using their ORCID identifiers, gaining access to a personal dashboard where they can create, edit, and submit annotations to RepeatsDB. All these annotations remain on hold until an expert curator reviews and approves them for publication in the next periodic release.

Biocurators can manually define or adjust the boundaries of repeat regions, units, and insertions, as well as assign the appropriate classifications. To facilitate this, the tool incorporates the same components (structure and sequence viewer) used throughout the database interface. Both are updated to reflect changes in units, regions, and insertions annotated through the main table, located in the bottom right side of the page. Most importantly, each repeat region is associated with a specific branch of the classification hierarchy (Class → Topology → Fold → Clan), and each unit and insertion is linked to a specific repeat region. Once the curation is complete, the user submits it for review.

#### Data curation on experimental structures

The current version of RepeatsDB includes 15 141 manually curated STRPs based on experimental structures from the PDB, covering a total of 4092 distinct UniProt protein sequences. This represents a nearly threefold increase compared to version 3.0, published in 2022. This achievement reflects a substantial human effort dedicated to ensuring the highest quality and continuous expansion of our reviewed entries. To achieve this goal, RepeatsDB was a key resource in the REFRACT project, a Marie Skłodowska-Curie Research and Innovation Staff Exchange (RISE) Horizon 2020 consortium funded by the European Union (URL: http://refract-rise.eu). This project involved institutions from Europe and Latin America, focusing on understanding tandem repeat proteins and establishing a standardized framework for their classification and annotation. Throughout the project, many seconded staff members from Latin American institutions made significant contributions to the curation of the RepeatsDB database.

The overall curation process was divided into two phases. The first phase involved a thorough review of all curated data from RepeatsDB version 3.0 to check inconsistencies and apply uniform criteria to all reviewed entries. This initial phase was carried out by expert curators with specialized knowledge in the biology and structural aspects of STRPs. The second phase consisted of running STRPsearch on the PDB to target the curation of those PDB entries that map to UniProt protein sequences not yet covered in RepeatsDB. The new version ensures that there is at least one curated experimental protein structure per protein sequence mapping to the PDB.

#### AlphaFoldDB entries

**Figure 1.**
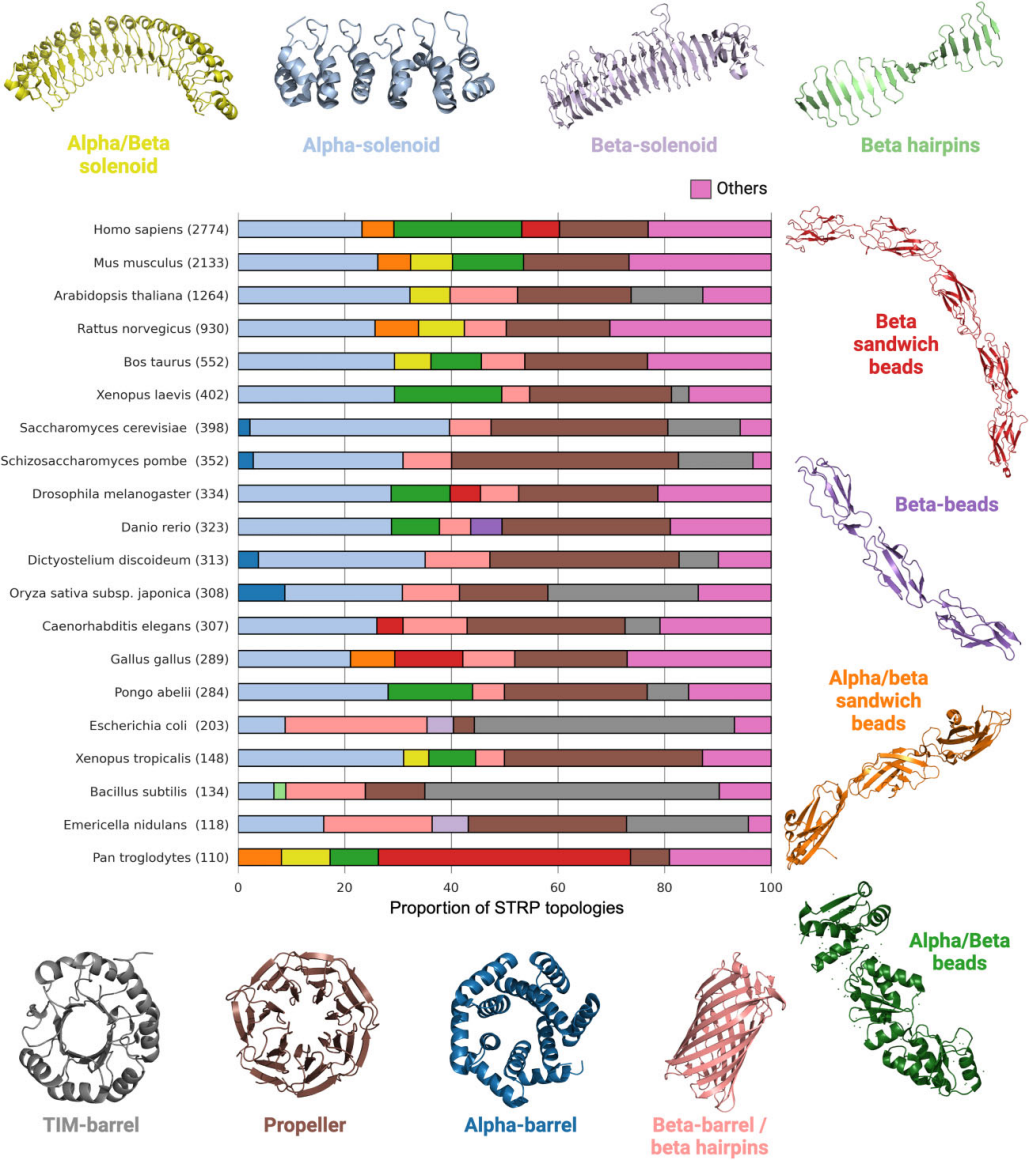
Proportion of STRP topologies identified in the top 20 most frequently occurring organisms in AlphaFoldDB/SwissProt models.

In this release, RepeatsDB included potential STRPs detected on AlphaFold structural models deposited in AlphaFoldDB. To accomplish this, STRPsearch was used to analyze the AlphaFold models of 542 378 protein structures in the SwissProt database. The parameters of STRPsearch were set to identify STRPs with an E-value threshold below 10^−5^, while all other parameters were left at their default values. This analysis resulted in the identification of 30220 unique proteins from 2598 different organisms, containing 33 207 repeat regions and 250 416 repeat units. Figure 1 displays the top 20 organisms based on the number of predicted STRPs, along with the different topologies represented within the total STRPs for each organism.

### Web site

RepeatsDB data is now accessible through both the website and a dedicated server API. Key improvements in this latest RepeatsDB release include: (i) enhancements to the API, which now offer improved performance with pagination and a RESTful architecture, ensuring scalability to handle the growing number of entries; (ii) a redesigned entry page that more effectively highlights repeat regions, associated Pfam domains, external annotations from InterPro and structural classifications; (iii) a new statistics page that allows users to explore database metrics based on repeat classifications and (iv) more streamlined browse and search interface that improves data searchability and accessibility, making it easier for users to find specific entries.

#### Browsing and searching data

In the new release of RepeatsDB, the main way of filtering a specific subset of annotations is through the browse/search page. It presents a search form at the top that allows filters for protein structure id, chain and source database. Recognized source databases are currently the PDB and the AlphaFoldDB. Moreover, it allows one to search for the type of annotation: whether this is a manually curated or an automatically predicted one by clicking on the top buttons, namely ‘reviewed’ and ‘predicted’, which work as checkboxes. Moreover, the interface allows searching for region classification and Pfam domain. In both those fields more than one value can be specified by clicking on the add button, or by pressing enter while focusing the input field. Each field performs a logical *or* operation: in case at least one searched term is found in an annotation, it is returned. Additionally, when the user types the class number or hovers over the ‘Region Classification’ field, a list of the entire classification tree appears. The list is automatically filtered based on the user's input.

Another way of accessing the search page is through the home page or the search box on the right corner of the upper navigation bar. In both cases a text form is presented: if the searched text matches exactly a UniProtKB accession number or a PDB structure and chain identifier, it will automatically attempt to redirect the user to the associated entry. Otherwise, it will redirect the user to the search page, where the form will be partially compiled with the previously entered information.

It is worth mentioning that the classification tree on the home page is clickable. Thus, by clicking on one of its nodes the user will be redirected to the search page. In this case, the search form will be configured to search only for regions in the selected classification branch.

#### Entry page

The entry page starts with a summary of essential information, including the positions of repeated regions, their classifications, the corresponding UniProt, Pfam and InterPro accessions with cross-links and boundaries for each annotation, as well as the Gene Ontology terms. The next section features an interactive structure viewer, accompanied by detailed information for each region displayed in table form. In the table, rows representing regions are colored violet, repeated units appear in alternating red and blue patterns, and insertions are highlighted in yellow. The table details the start and end positions of repeat regions, units, and insertions, as well as the corresponding region classification or parent region for units and insertions.

**Figure 2.**
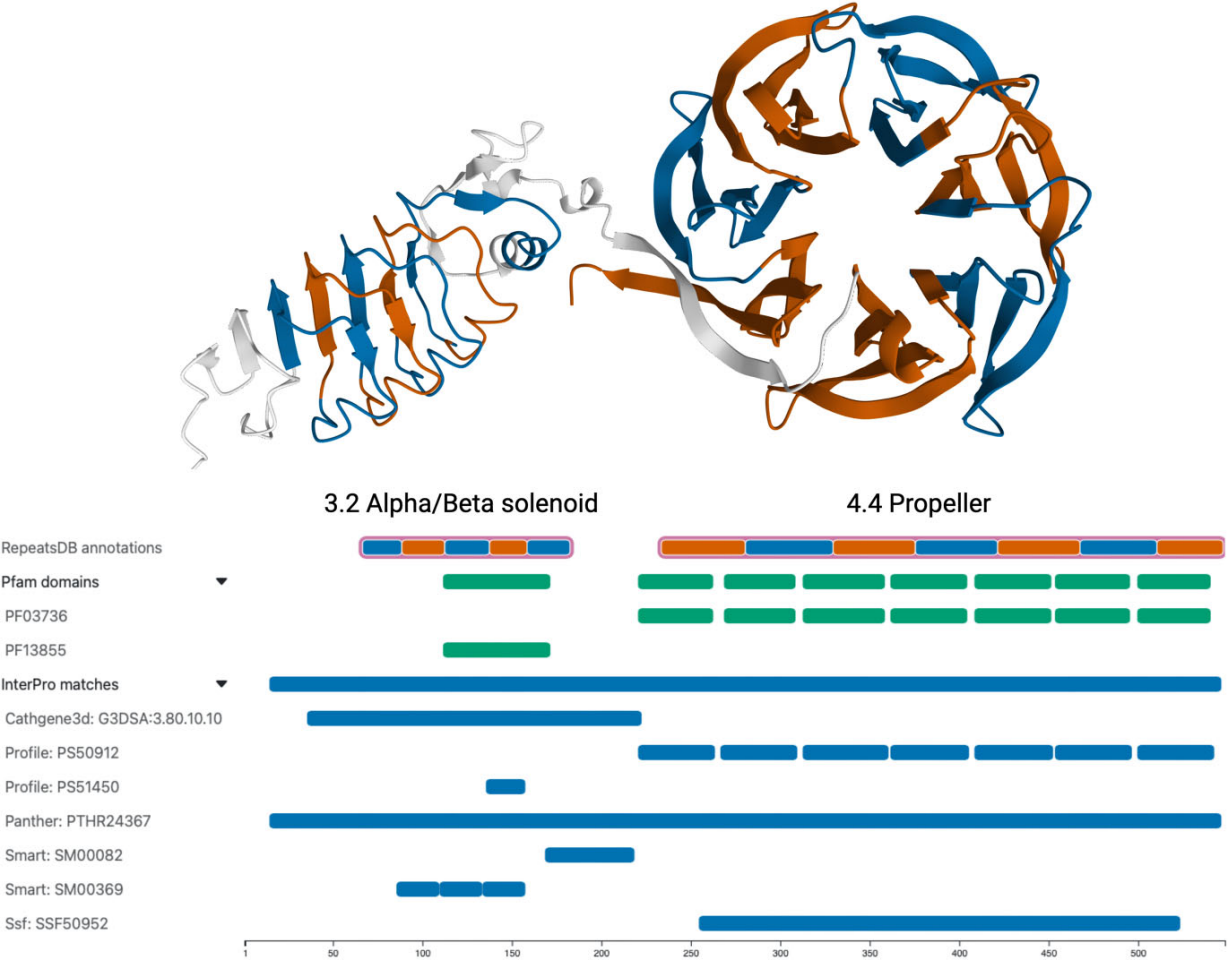
AlphaFold model of the human leucine-rich repeat LGI family member 3 (UniProt accession: Q8N145). The feature viewer displays all information mapped onto the structure's reference sequence. The top track shows two distinct predicted repeat regions in this protein: a 3.2 alpha/beta solenoid and a 4.4 propeller, with repetitive units colored in alternating blue and red. The tracks below display various protein sequence features, including Pfam domains and InterPro annotations.

The next section of the page includes a protein feature viewer, which allows users to view different sequence features from multiple sources in a single visualization (Figure 2). The ‘RepeatDB annotations’ track displays repeat regions, units, and insertions (if present) mapped on the sequence from the source structure, either PDB or AlphaFoldDB. The other two tracks display external features. For PDB structures, UniProt coverage is shown in blue; for AlphaFold models, InterPro coverage is shown in blue. Pfam coverage is displayed in green for both. These tracks can be expanded into sub-tracks for visualizing the accession numbers. All tracks are zoomable using the mouse wheel or by selecting a specific region.

In the bottom section, users can select a region using the violet buttons to access detailed sequence and structural analyses of its units. Below this, a breakdown of all classifications annotated for the entry and a brief description are provided. Multiple sequence alignment is conducted using Clustal Omega software with default parameters. The alignment is displayed in a sequence viewer, where residues are colored based on their physicochemical properties using the Jalview Zappo color scheme. Additionally, a sequence similarity matrix is provided to visualize the similarities between each pair of unit sequences. Multiple structural alignments are performed using TM-align (29) with default parameters. The displayed results include the superimposed unit structures and a matrix displaying TM-scores on the upper diagonal and root mean square deviation (RMSD) on the lower between units. Additionally, a sequence viewer shows the sequence alignment derived from the structural alignment. All this information can be easily downloaded in several formats by clicking the blue buttons next to the corresponding titles.

### Server and API

RepeatsDB features a novel architecture that integrates data storage and distribution capabilities. The architecture is divided into two partitions: staging and production. The staging partition handles biocuration activities, allowing annotations to be created, updated, and deleted by users. These annotations contain only the minimal information required to describe themselves. The production partition manages consolidated and enriched data, which has been processed using external resources and third-party software. Data flows into the production partition through a specific update pipeline executed before each public release of the database. This pipeline performs structural and multiple sequence alignments among units, calculates TM-Score and RMSD matrices, and maps regions annotated on PDB sequences to their corresponding UniProt sequences using SIFTS (30).

Annotations are described by the Annotation Graph library. Although based on a NoSQL database, the library adopts a graph-like data structure where nodes represent generic biological annotations. This architecture allows the staging partition to remain online without shutting down the library implementation when releasing a new production version. Moreover, it decouples bio-curation efforts, ensuring that individual users are unaware of others' activities. A limited group of trusted users, referred to as reviewers, selects annotations suitable for the next release and flags them as reviewed. Once an annotation (node) is reviewed, it becomes immutable; to modify or delete its content, a child node must be created. This approach ensures that specific snapshots of the staging partition can always be reproduced.

The Annotation Graph library is included with RepeatsDB. Its implementation has three levels: the front end, the back end, and the database. The front end is based on the Angular framework. The back end is developed using NestJS over the Express framework. The database is a MongoDB instance. In RepeatsDB, there are two different front-end applications: (i) RepeatsDB Bio-Curation Tool: handles staging data, allowing entries in the database to be created, modified, or deleted; (ii) RepeatsDB User Interface: allows users to browse through production data. Both applications communicate with the same back end, which in turn communicates with two different databases within the same MongoDB instance, implementing staging and production partitioning. To achieve this, ngx-mol-viewers package (URL: https://biocomputingup.github.io/ngx-mol-viewers/) was developed to provide a collection of modular and reusable components easily integrated into any Angular-based web application. This package supports the sequence, structure, and feature viewers used in the RepeatsDB UI and Bio-Curation tool.

The Swagger documentation page for the API (URL: https://dev.repeatsdb.org/api/) provides a comprehensive overview of the available endpoints, operations, and data models for interacting with the RepeatsDB API. The page is structured in a user-friendly manner, with a navigation bar on the left-hand side that allows you to browse through different sections of the documentation. The main content area displays detailed information about each API endpoint, including its path, HTTP methods (such as GET, POST, PUT, DELETE), and a brief description of its purpose.

## Conclusions and future work

Understanding STRPs is crucial due to their extensive roles in biological systems and their potential for biotechnological applications. STRPs are fundamental not only to numerous cellular functions but also represent a rich domain for therapeutic interventions and biomaterial design. Their unique structural features and dynamic roles in cell signaling, molecular recognition, and self-assembly processes make them a focal point for advanced research in protein science. RepeatsDB serves as the primary repository for annotating and classifying these proteins. This new version significantly advances the curation and classification of STRPs, facilitating integration with broader structural protein data repositories. The combination of manual and automated curation processes, supported by innovative tools such as STRPsearch and the Bio-Curation tool, has enabled a comprehensive update and expansion of the database. RepeatsDB was the core resource of the REFRACT consortium and is crucial for the efforts pursued by the COST Action ‘Machine Learning for Non-Globular Proteins’ (ML4NGP).

Looking ahead, RepeatsDB plans to integrate with APICURON (31), aiming to recognize and reward the efforts of biocurators. This integration will foster a more engaged and active community around STRP data curation, which is particularly crucial as we extend our efforts to include AlphaFold models from TrEMBL. Additionally, we will continue to enhance our classification schemas and refine repeat definitions in collaboration with the InterPro consortium. These initiatives will ensure that RepeatsDB remains a leading resource in the detection and classification of STRPs, providing essential support for researchers worldwide.

## Data Availability

All the data is available from the URL: https://repeatsdb.org.

## Appendix

### RepeatsDB curators

Estefanía Lorena Borucki^5^, Maia Cabrera^6^, Patricio Chinestrad^6^, Ian Czarnowski^6^, Jose Francisco Lombardo^7^, Pablo Lorenzano Menna^6^, Ezequiel Gerardo Mogro^8^, Carla Luciana Padilla Franzotti^9^, Julia Yamila Santillan^9^

^5^Laboratorio de Biotransformaciones y Química de Ácidos Nucleicos, Departamento de Ciencia y Tecnología, Universidad Nacional de Quilmes, Argentina

^6^Laboratorio de Farmacología Molecular, Departamento de Ciencia y Tecnología, Universidad Nacional de Quilmes, Argentina

^7^Instituto de Investigaciones Bioquímicas de La Plata (INIBIOLP), Facultad de Ciencias Médicas, Universidad Nacional de La Plata (UNLP)-Consejo Nacional de Investigaciones Científicas Y Técnicas (CONICET), La Plata, Argentina

^8^Instituto de Biotecnología y Biología Molecular (IBBM), CONICET, CCT-La Plata, Universidad Nacional de La Plata (UNLP), Argentina

^9^Department of Science and Technology, National University of Quilmes-CONICET, Bernal, Argentina
